# Mixed convection flow along a curved surface in the presence of exothermic catalytic chemical reaction

**DOI:** 10.1038/s41598-021-92409-3

**Published:** 2021-06-18

**Authors:** Uzma Ahmad, Muhammad Ashraf, Amir Abbas, A. M. Rashad, Hossam A. Nabwey

**Affiliations:** 1 Department of Mathematics, Faculty of Science, University of Sargodha, Sargodha, 40100 Pakistan; 2 Department of Mathematics, Faculty of Science, Aswan University, Aswan, 81528 Egypt; 3 Department of Mathematics, College of Science and Humanities in Al-Kharj, Prince Sattam Bin Abdulaziz University, Al-Kharj, 11942 Saudi Arabia; 4 Department of Basic Engineering Science, Faculty of Engineering, Menoufia University, Shebin El-Kom, 32511 Egypt

**Keywords:** Engineering, Mathematics and computing, Physics

## Abstract

In the current study, the attention is paid on the phenomena of mixed convection flow under the effect of exothermic catalytic chemical reaction along the curved surface. The proposed problem is modeled in nonlinear coupled partial differential equations. In keeping view the principle of homogeneity the dimensional flow model is transformed into dimensionless by using an appropriate scaling. This well arranged form of equations is then discretized with the aid of finite difference method for the numerical solution. The solutions of the considered model are estimated and displayed in the graphs. Here, in the contemporary study variables of physical significance such as velocity profile, temperature distribution and mass concentration are encountered efficiently. The incorporated pertinent dimensionless numbers that is body shape parameter, mixed convection parameter, modified mixed convection parameter, Prandtle number, exothermic parameter, chemical reaction parameter, temperature relative parameter, dimensionless activation energy parameter, and Schmidt number for which variations in the concentrated physical variables are estimated and presented in graphical way. For each boundary conditions computations are performed along the curved surface for different body shape parameter (n) values range from 0 up to 0.5; the obtained results satisfied by the boundary conditions. The velocity profile becomes increasingly more significant for n equal to 1 and due to the uniformly heated surface temperature profile and mass concentration are uniformly distributed.

## Introduction

Heat transfer process is the process which is concerned with conversion and exchange of thermal energy and heat between the systems. Heat transfer is classical field into various mechanisms such as thermal convection, transfer of energy by phase changes, thermal convection, and thermal radiation. The transfer of heat occurs due to bulk movement of the fluid (liquid and gasses) which carries along them with flow of matter in the fluid. On the other side mass transfer is collective movement of the mass form one location, usually meaning stream, fraction or components phase changes to one another. Mass transfer occurs in many processes such as evaporation, drying, absorption, precipitation, membrane filtration and distillation. The combined phenomena of heat and mass transfer occur in engineering for physical processes which are concerned with diffusive and convective transport of chemical species. The term of Arrheniuous activation energy was introduced by Svante Arrhenius in 1889.This is the energy which is required to produce the chemical reaction with potential reactants. Exothermic chemical reactions are the processes which require or give off. Highly exothermic chemical reactions are needed to thrust spacecraft into air. White plumes following the craft are reaction produce gasses dispersing aluminum oxide. The combination of mixed convection flow and catalytic chemical reactions has so many applications in engineering especially in chemical engineering, thermal engineering and industry. The above said mechanisms attracted much attention of the researchers on different flow geometries and flow conditions.

Pop and Thakar^1^ encountered the phenomena of free convection flow over the curved surface theoretically. A note on mechanism of self-similar natural convection flow from a heated curved surface has been considered by Magyari et al.^2^. Maleque^3^ focused on magnetohydrodynamics natural convection flow numerically. He included the effects of radiation, exothermic/endothermic chemical reaction, activation energy and magnetic field simultaneously in his study. Similarity solutions of mixed convection flow along a vertical plate with slip flow conditions are computed by Krishnendu et al.^4^. Bachok et al.^5^ investigated numerically the mixed convection flow along the vertical flat plate in the external fluid flow considering the effects of viscous dissipation. Hayat et al*.*^6^ proposed the novel study of Marangoni mixed convection flow coupled with radiation and Joule heating effects. They also took into account the impacts of inclined magnetic field and viscous dissipation impacts. MHD mixed convection flow along the vertical plate with convective boundary conditions along with heat source and chemical has analyzed by Rajeswari and Manjum^7^. The numerical investigations on unsteady mixed convection flow along the surface of a sphere with dissipation effects have been studied by Ashraf et al*.*^8^. Okehi et al.^9^ addressed the boundary layer flow induced by exponentially stretched curved surface in the presence of dissipation effects. They solve the self-similar equations by using shooting and R–K methods.

Theoretical analysis on boundary layer flow along the curved stretching surface under the convective boundary conditions of heat and mass transfer has been conducted by Hayat el al.^10^. Computational treatment of natural convection flow along the curved surface in the presence of catalytic chemical reaction has been done by Ashraf et al*.*^11^. Unsteady mixed convection flow along the infinite length with the combined impacts of activation energy and binary chemical reaction has been addressed by Dhlamini et al.^12^ and highlighted the influences of thermophoresis, Brownian motion and viscous dissipation. Discussion on mixed convection flow coupled with and thermophoresis phenomena along with the effects of temperature dependent viscosity, thermal conductivity, and heat generation effects along the surface of a sphere has been conducted in^13–16^. The fluid flow of nanofluid due to rotating disk with the consideration of activation energy and magnetic field has been addressed in by Alghamdi^17^. Uzma and Ashraf^18^ tackled the problem of natural convection flow under the impact of exothermic catalytic chemical reaction along with the viscous dissipation effects on curved surface. Zia et al.^19^ have taken in to account the problem of mixed convection oscillatory flow along the non-conducting cylinder under the influence of thermal stratification. Chudhary and Kanika^20^ studied the phenomena of nanofluid flow along the particle shape over exponential temperature with the effect of thermal radiation, magnetic field and viscous dissipation. Heat and mass transfer analysis of MHD flow through porous medium along infinite plate has been conducted by Krishna et al*.*^21^. Saeed et al.^22^ focused their attention on nanofluid flow along the curved surface in the presence of magnetic field, chemical reaction and Arrhenius activation energy. Muhammad et al*.*^23^ proposed the model of fully developed flow Darcy–Forchheimer mixed convection flow along the curved surface in the presence of activation energy and entropy generation. Khan et al*.*^24^ investigated theoretically the phenomena of mixed convection flow equipped with entropy optimization, electric field, magnetic field and second order slip conditions. The computational study of heat transfer in mixed convection flow of blood based carbon nanotubes over the curved stretching surface has been conducted by Hayat et al*.*^25^.

Being motivated from the above said applications and literature review the current study is concerned with the mixed convection flow coupled with the exothermic catalytic chemical reaction along a curved surface. In the coming sections the mathematical model for the proposed problem will be established in terms of nonlinear partial differential equations and then the developed model will besolved by using finite difference method. The obtained numerical results in terms of the effects of different parameters on velocity profile, temperature distribution, mass concentration, skin friction, heat and mass transfer are displayed graphically. The novelty of the current work is to highlight the behavior above mentioned quantities along the curved surface.

## Statement of the problem and mathematical formulation mathematical model and solution methodology

**Figure 1:**
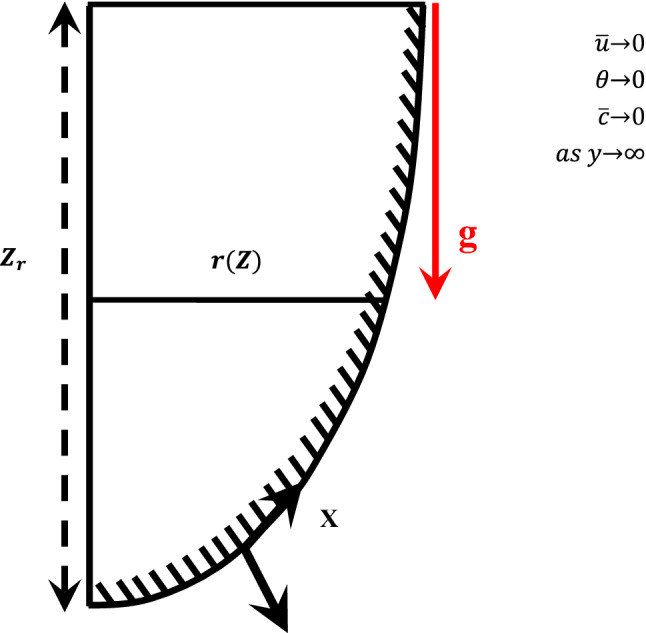
Coordinate system and flow configuration of the proposed model.

Consider steady, two dimensional, incompressible and mixed convection fluid flow along the curved surface in the presence of exothermic chemical reaction. The horizontal axis on the curved surface is assumed along x-axis and normal axis is taken along y-axis. The velocity components along horizontal and normal axis are u and v respecively. The graphical vision of the problem and the coordinate system of such a flow model are manifested in Fig. 1. The dimensionless flow model in terms of system of the coupled partial differential equations given as below:

1 $$\frac{\partial \stackrel{-}{u}}{\partial \stackrel{-}{x}}+\frac{\partial \stackrel{-}{v}}{\partial \stackrel{-}{y}}$$

2 $$\stackrel{-}{u}\frac{\partial \stackrel{-}{u}}{\partial \stackrel{-}{x}}+\stackrel{-}{v}\frac{\partial \stackrel{-}{u}}{\partial \stackrel{-}{y}}+\frac{{\stackrel{-}{u}}^{2}}{2x}\left(P(\stackrel{-}{x})+Q(\stackrel{-}{x})\right)=\frac{{\partial }^{2}\stackrel{-}{u}}{\partial {\stackrel{-}{y}}^{2}}+{\lambda }_{T}\theta +{\lambda }_{C}\phi$$

3 $$\stackrel{-}{u}\frac{\partial \theta }{\partial \stackrel{-}{x}}+\stackrel{-}{v}\frac{\partial \theta }{\partial \stackrel{-}{y}}=\frac{1}{Pr}\frac{{\partial }^{2}\theta }{\partial {\stackrel{-}{y}}^{2}}+\beta {\lambda }^{2}\left(1+n\gamma \theta \right)\theta {e}^{\frac{-E}{1+\gamma \theta }}$$

4 $$\stackrel{-}{u}\frac{\partial \phi }{\partial \stackrel{-}{x}}+\stackrel{-}{v}\frac{\partial \phi }{\partial \stackrel{-}{y}}=\frac{1}{Sc}\frac{{\partial }^{2}\phi }{\partial {\stackrel{-}{y}}^{2}}+{\lambda }^{2}\left(1+n\gamma \theta \right)\phi {e}^{\frac{-E}{1+\gamma \theta }}$$

Dimensionless form of boundary conditions satisfied by the system of equations given in (1)–(4)

5 $$\begin{gathered} \bar{u} = 0, \quad \bar{v} = 0, \quad \theta = 1, \quad \phi = 1 \quad at \quad \bar{y} = 0 \hfill \\ \bar{u} \to 1, \quad \hat{\theta } \to 0, \quad \phi \to 0 \quad as \quad \bar{y} \to \infty . \hfill \\ \end{gathered}$$

These are the dimensionless variables used in the above equations

6 $$\begin{aligned} & \bar{x} = \frac{x}{l},\quad \bar{y} = \frac{y}{l}Re_{L} ^{{{\raise0.7ex\hbox{$1$} \!\mathord{\left/ {\vphantom {1 2}}\right.\kern-\nulldelimiterspace} \!\lower0.7ex\hbox{$2$}}}} ,\quad \bar{u} = \frac{u}{{U_{s} }},\quad \bar{v} = \frac{v}{{U_{\infty } }}Re_{l} ^{{{\raise0.7ex\hbox{$1$} \!\mathord{\left/ {\vphantom {1 2}}\right.\kern-\nulldelimiterspace} \!\lower0.7ex\hbox{$2$}}}} , \\ & \theta = \frac{{T - T_{\infty } }}{{T_{w} - T_{\infty } }},\quad \phi = \frac{{C - C_{\infty } }}{{C_{w} - C_{\infty } }}. \\ \end{aligned}$$

We also define the velocity scale, Grashof number and Reynolds number as

$$\begin{aligned} & \Delta T = T_{w} - T_{\infty } ,\quad \Delta C = C_{w} - C_{\infty } ,\quad U_{s} = \left( {g_{x} \beta \Delta Tl} \right)^{{{\raise0.7ex\hbox{$1$} \!\mathord{\left/ {\vphantom {1 2}}\right.\kern-\nulldelimiterspace} \!\lower0.7ex\hbox{$2$}}}} ,\quad Gr = \frac{{g_{x} \beta \Delta Tl^{3} }}{{\nu ^{2} }}, \\ & Gr^{*} = \frac{{g_{x} \beta \Delta Cl^{3} }}{{\nu ^{2} }},\quad Re_{l} = \frac{{U_{s} l}}{\nu } \\ \end{aligned}$$

In the above equations, ${\lambda }_{T}=\frac{{Gr}_{l}}{{Re}_{l}^{2,}}$ is the mixed convection parameter, also known as, ${\lambda }_{C}=\frac{{Gr}_{l}^{*}}{{Re}_{l}^{2}}$ is the modified mixed convection parameter, $\beta =1$ is the exothermic. The symbol ${\lambda }^{2}=\frac{{k}_{r}^{2}l}{{U}_{s}}$ is the dimensionless chemical reaction rate constant,where ${k}_{r}^{2}$ is the chemical reaction rate constant, $l$ is the characteristic length. The notations $\gamma =\frac{{T}_{w}-{T}_{\infty }}{{T}_{\infty }}$ is the temperature relative parameter, $E=\frac{{E}_{a}}{k{T}_{\infty }}$ is the dimensionless activation energy, with ${E}_{a}$ as the activation energy and $k=1.380649\times {10}^{-23}$ JK^−1^ is the Boltzman constant. Moreover, Pr = $\frac{\nu }{\alpha }$ is the Prandtl number and Sc = $\frac{\nu }{{D}_{m}}$ is the Schmidt number respectively.

Also $P(\stackrel{-}{x})$, the wall temperature function and the body shape function $Q(\stackrel{-}{x})$ are defined as (see detail^5^)

7 $$P\left( {\bar{x}} \right) = \frac{{dlnT_{s} }}{{dlnxl}},\quad Q\left( {\bar{x}} \right) = \frac{{dlng_{x} }}{{dlnxl}}$$

where It is important to point out that the system of partial differential equations given in (1)–(4) formulates a general mathematical form which is suitable for mixed convection flow over heated body of an arbitrary curved shape in the presence of exothermic catalytic chemical reaction. For the special case in which both $P(x)$ and $Q(x)$ are constants namely, $m$ and $n$, which satisfy the relation $m+n=1$ (see^5^). Thus we have the following power functions along $x$-axis of the surface temperature and the tangential component of acceleration

8 $$g_{x} \sim x^{n} ,\quad \Delta T\sim ~x^{m}$$

Under the conditions given in (7) and (8), the conservation Eqs. (2)–(4) along with boundary conditions (5) (by dropping bars) take the following form

9 $$\frac{\partial u}{\partial x}+\frac{\partial v}{\partial y}=0,$$

10 $$u\frac{\partial u}{\partial x}+v\frac{\partial u}{\partial y}+\stackrel{\sim }{n}\frac{{u}^{2}}{2x}=\frac{{\partial }^{2}u}{\partial {y}^{2}}+{\lambda }_{T}\theta +{\lambda }_{C}\phi ,$$

11 $$u\frac{{\partial \theta }}{{\partial x}} + v\frac{{\partial \theta }}{{\partial y}} = \frac{1}{{Pr}}\frac{{\partial ^{2} \theta }}{{\partial y^{2} }} + \beta \lambda ^{2} \left( {1 + n\gamma \theta } \right)\theta e^{{\frac{{ - E}}{{1 + \gamma \theta }}}} ,$$

12 $$u\frac{\partial \phi }{\partial x}+v\frac{\partial \phi }{\partial y}=\frac{1}{Sc}\frac{{\partial }^{2}\phi }{\partial {y}^{2}}+{\lambda }^{2}\left(1+n\gamma \theta \right)\phi {e}^{\frac{-E}{1+\gamma \theta }}$$

Subject to the boundary conditions

13 $$\begin{aligned} & u = 0,\quad v = 0,\quad \theta = 1,\quad \phi = 1\quad at\quad y = 0 \\ & u \to 1,\quad \theta \to 0,\quad \phi \to 0\quad as\quad y \to \infty . \\ \end{aligned}$$

### Primitive variable formulation

To create the straight forwardness in the above equations for numerical algorithm on computer, we use the following transformation variables known as primitive variable formulation. The primitive variables formulation for dependent and independent variables is given below:

14 $$u = U\left( {X,Y} \right),\quad v = x^{{{\raise0.7ex\hbox{${ - 1}$} \!\mathord{\left/ {\vphantom {{ - 1} 2}}\right.\kern-\nulldelimiterspace} \!\lower0.7ex\hbox{$2$}}}} V\left( {X,Y} \right),\quad x = X,\quad y = x^{{{\raise0.7ex\hbox{$1$} \!\mathord{\left/ {\vphantom {1 2}}\right.\kern-\nulldelimiterspace} \!\lower0.7ex\hbox{$2$}}}} Y,\quad \theta = \Theta \left( {X,Y} \right),\quad \phi = \Phi \left( {X,Y} \right)$$

By using Eq. (14) into Eqs. (9)–(12), we have

15 $$X\frac{{\partial U}}{{\partial X}} - \frac{Y}{2}\frac{{\partial U}}{{\partial Y}} + \frac{{\partial V}}{{\partial Y}} = 0,$$

16 $$\frac{n}{2}U^{2} + XU\frac{{\partial U}}{{\partial X}} + \left( {V - \frac{{YU}}{2}} \right)\frac{{\partial U}}{{\partial Y}} = \frac{{\partial ^{2} U}}{{\partial Y^{2} }} + \lambda _{T} \Theta + \lambda _{C} \Phi ,$$

17 $$XU\frac{{\partial \Theta }}{{\partial X}} + \left( {V - \frac{{YU}}{2}} \right)\frac{{\partial \Theta }}{{\partial Y}} = \frac{1}{{Pr}}\frac{{\partial ^{2} \Theta }}{{\partial Y^{2} }} + \beta \lambda ^{2} \left( {1 + n\gamma \Theta } \right)\left( {1 - E + E\gamma \Theta } \right)\Theta ,$$

18 $$XU\frac{{\partial \Phi }}{{\partial X}} + \left( {V - \frac{{YU}}{2}} \right)\frac{{\partial \Phi }}{{\partial Y}} = \frac{1}{{Sc}}\frac{{\partial ^{2} \Phi }}{{\partial Y^{2} }} + \lambda ^{2} \left( {1 + n\gamma \Theta } \right)\left( {1 - E + E\gamma \Theta } \right)\Phi .$$

The transformed boundary conditions are

19 $$\begin{aligned} & U = 0,\quad V = 0,\quad \Theta = 1,\quad \Phi = 1\quad at\quad Y = 0 \\ & U \to 1,\quad \Theta \to 0,\quad \Phi \to 0\quad as\quad Y \to \infty \\ \end{aligned}$$

### Discretization

The above system of transformed boundary layer equations are solved with application of finite difference method. With the use of finite difference method the differential equations are transformed into algebraic system of equations which are kwon as discretized form of equations

20 $$\frac{{\partial U}}{{\partial X}} = \frac{{U_{{i,j}} - U_{{i,j - 1}} }}{{\Delta X}},\quad \frac{{\partial U}}{{\partial Y}} = \frac{{U_{{i + 1,j}} - U_{{i - 1,j}} }}{{2\Delta Y}},\quad \frac{{\partial ^{2} U}}{{\partial Y^{2} }} = \frac{{U_{{i - 1,j}} - 2U_{{i,j}} + U_{{i + 1,j}} }}{{\Delta Y^{2} }}$$

21 $${V}_{i+1,j}={V}_{i-1,j}-2\frac{\Delta Y}{\Delta X}{X}_{i}\left({U}_{i,j}-{U}_{i,j-1}\right)+\frac{{Y}_{j}}{2}\left({U}_{i+1,j}-{U}_{i-1,j}\right),$$

Momentum equation

22 $$A_{1} U_{{i - 1,j}} + B_{1} U_{{i,j}} + C_{1} U_{{i + 1,j}} = D_{1} ,$$

where

$${A}_{1}=1+\frac{\Delta Y}{2}\left({V}_{i,j}-\frac{{U}_{i,j}{Y}_{j}}{2}\right),$$

$${B}_{1}=\left[\frac{1}{2}-\frac{{X}_{i}}{\Delta X}\right]\Delta {Y}^{2}{U}_{i,j}-2,$$

$${C}_{1}=1-\frac{\Delta Y}{2}\left({V}_{i,j}-\frac{{U}_{i,j}{Y}_{j}}{2}\right),$$

$$D_{1} = - \frac{{X_{i} }}{{\Delta X}}\Delta Y^{2} U_{{i,j}} U_{{i,j - 1}} - \Delta Y^{2} \left( {\lambda _{T} \Theta _{{i,j}} + \lambda _{C} \Phi _{{i,j}} } \right).$$

Energy equation

23 $$A_{2} \theta _{{i - 1,j}} + B_{2} \theta _{{i,j}} + C_{2} \theta _{{i + 1,j}} = D_{2} ,$$

where

$${A}_{2}=\frac{1}{Pr}+\frac{\Delta Y}{2}\left({V}_{i,j}-\frac{{U}_{i,j}{Y}_{j}}{2}\right),$$

$$B_{2} = - \frac{2}{{Pr}} - \frac{{X_{i} }}{{\Delta X}}\Delta Y^{2} U_{{i,j}} + \beta X_{i} \lambda ^{2} \Delta y^{2} \left( {1 + n\gamma \Theta _{{i,j}} } \right)\left( {1 - E + E\gamma \Theta _{{i,j}} } \right),$$

$${C}_{2}=\frac{1}{Pr}-\frac{\Delta Y}{2}\left({V}_{i,j}-\frac{{U}_{i,j}{Y}_{j}}{2}\right),$$

$$D_{2} = - \frac{{X_{i} }}{{\Delta X}}\Delta Y^{2} U_{{i,j}} \Theta _{{i,j - 1}}$$

Mass equation

24 $$A_{3} \Phi _{{i - 1,j}} + B_{3} \Phi _{{i,j}} + C_{3} \Phi _{{i + 1,j}} = D_{3} ,$$

where

$${A}_{3}=\frac{1}{Sc}+\frac{\Delta Y}{2}\left({V}_{i,j}-\frac{{U}_{i,j}{Y}_{j}}{5}\right),$$

$$B_{3} = - \frac{2}{{Sc}} - \frac{{{\text{X}}_{{\text{i}}} }}{{\Delta X}}\Delta Y^{2} U_{{i,j}} + X_{i} \lambda ^{2} \Delta y^{2} \left( {1 + n\gamma \Theta _{{i,j}} } \right)\left( {1 - E + E\gamma \Theta _{{i,j}} } \right),$$

$${C}_{3}=\frac{1}{Sc}-\frac{\Delta Y}{2}\left({V}_{i,j}-\frac{{U}_{i,j}{Y}_{j}}{5}\right),$$

$$D_{3} = - \frac{{X_{i} }}{{\Delta X}}\Delta Y^{2} U_{{i,j}} \Phi _{{i,j - 1}} .$$

The discretized boundary conditions are

25 $$\begin{aligned} & U_{{i,j}} = 0,\quad {\text{V}}_{{{\text{i}},{\text{j}}}} = 0,\quad \theta _{{{\text{i}},{\text{j}}}} = 1,\quad \Phi _{{{\text{i}},{\text{j}}}} = 1\quad {\text{at}}\quad {\text{Y}}_{{\text{j}}} = 0 \\ & U_{{i,j}} \to 1,\quad \theta _{{i,j}} \to 0,\quad \Phi _{{i.j}} \to 0\quad as\quad Y_{j} \to \infty . \\ \end{aligned}$$

The foregoing governing equations together with the boundary conditions are solved numerically on a uniform mesh using Gauss elimination algorithm developed in previous study^18^. The discretization of the partial differential equations basically involved in second order central difference approximation for all diffusion terms and quick approximation for convective terms. The iterative calculations were continued until a relative convergence criterion of ${10}^{-5}$ was satisfied by all the field variables of the problem.

The obtained value of $\left( {\frac{{\partial \uptheta }}{{\partial {\text{Y}}}}} \right)_{{{\text{Y}} = 0}}$ is compared with Pop and Takhar^1^ in the presence of mass transfer to ensure the validity of the procedure. The comparison results are demonstrated in Table 1 and found to be in very good agreement.

**Table 1:** The comparison of obtained results for $\left( {\frac{{\partial \uptheta }}{{\partial {\text{Y}}}}} \right)_{{{\text{Y}} = 0}}$ by the present result and Pop and Takhar^1^ for Pr = 1.0 by keeping other parameters constant.

*n*	Present	Pop and Takhar^1^
0.1	0.3618	0.3690
0.2	0.3457	0.3469
0.3	0.2944	0.2949
0.4	0.24107	0.2488
0.5	0.19203	0.1946

## Results and discussion

**Figure 2:**
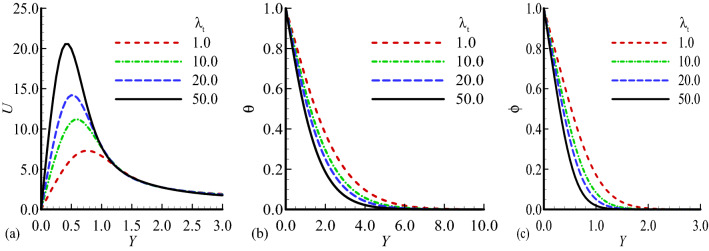
Dimensionless (**a**) Velocity U, (**b**) Temperature $\theta$ and (**c**) mass distribution $\phi$ for different values of ${\lambda }_{T}=1.0,$ 10.0, 20.0, 50.0 while $n=0.3,$ $E=0.2$, $\gamma =0.1$, $\lambda =0.5$, $\beta =0.5$, ${\lambda }_{c}=20$, Pr = 7.0, Sc = 0.6.

**Figure 3:**
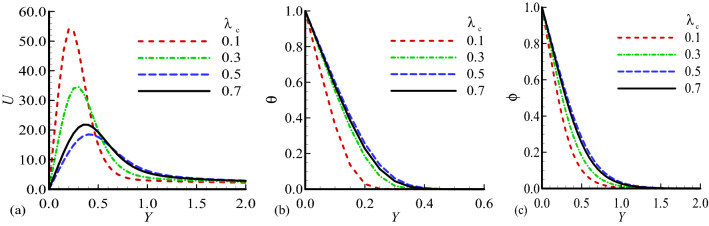
Dimensionless (**a**) Velocity $U$, (**b**) temperature $\theta$ and (**c**) mass distribution $\phi$ for different values of ${\lambda }_{c}=0.1,$ 0.3, 0.5, 0.7 while $n=0.3$, $E=0.2$, $\gamma =0.1$, $\lambda =0.5$, $\beta =0.5$, ${\lambda }_{T}=20$, Pr = 7.0, Sc = 0.6.

**Figure 4:**
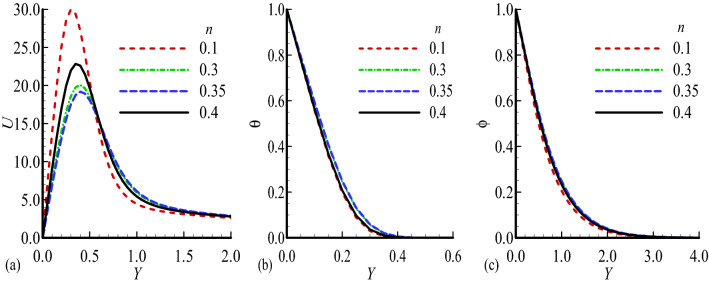
Dimensionless (**a**) Velocity $U$, (**b**) temperature $\theta$ and (**c**) mass distribution $\phi$ for different values of $n=0.1,$ 0.3, 0.35, 0.4 while $E=0.5$, $\gamma =0.1$, $\lambda =0.5$, $\beta =0.3$, ${\lambda }_{T}=1.0$, ${\lambda }_{c}=0.4$, Pr = 7.0, Sc = 0.2.

**Figure 5:**
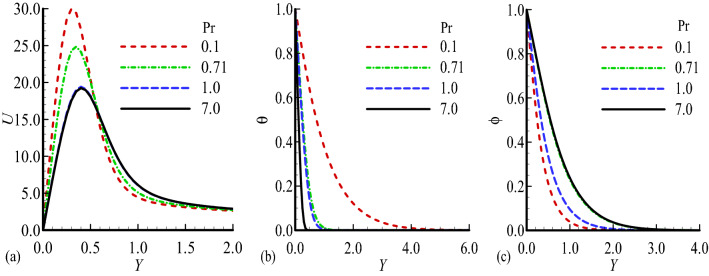
Dimensionless (**a**) Velocity $U$, (**b**) temperature $\theta$ and (**c**) mass distribution $\phi$ for different values of Pr $=0.1,$ 0.71, 1.0, 7.0 while $n=0.3$, $E=0.5$, $\gamma =0.1$, $\lambda =0.5$, $\beta =0.5$, ${\lambda }_{T}=15.0$, ${\lambda }_{c}=10.0,$ Sc = 0.2.

**Figure 6:**
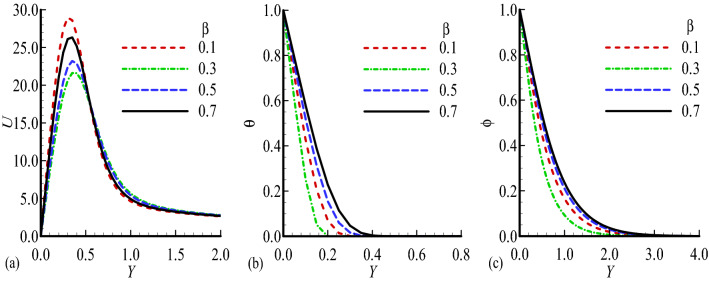
Dimensionless (**a**) Velocity $U$, (**b**) temperature $\theta$ and (**c**) mass distribution $\phi$ for different values of $\beta =0.1,$ 0.3, 0.5, 0.7 while $n=0.3$, $E=0.2$, $\gamma =0.2$, $\lambda =0.4$, ${\lambda }_{T}=10.0$, ${\lambda }_{c}=10.0,$ Pr = 7.0, Sc = 0.2.

**Figure 7:**
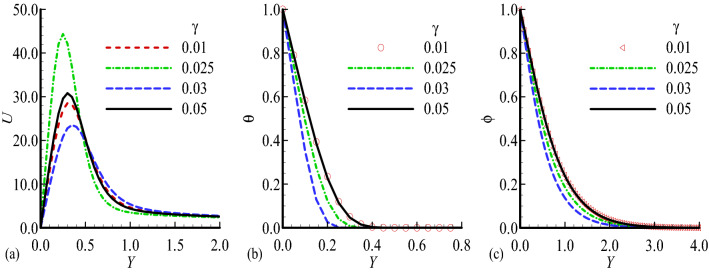
Dimensionless (**a**) Velocity $U$, (**b**) temperature $\theta$ and (**c**) mass distribution $\phi$ for different values of $\gamma =0.01,$ 0.25, 0.03, 0.05 while $n=0.3$, $E=0.8$, $\gamma =0.2$, $\lambda =0.5$, $\beta =0.5$, ${\lambda }_{T}=15.0$, ${\lambda }_{c}=10.0,$ Pr = 7.0, Sc = 0.2.

**Figure 8:**
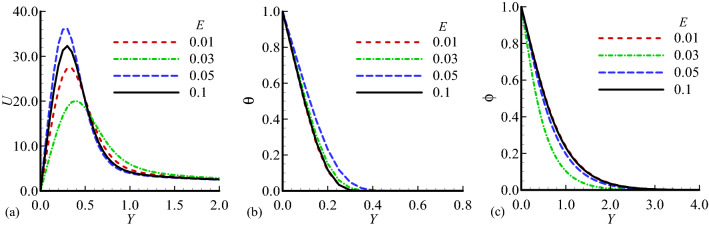
Dimensionless (**a**) Velocity $U$, (**b**) temperature $\theta$ and (**c**) mass distribution $\phi$ for different values of $E=0.01,$ 0.03, 0.05, 0.1 while $n=0.3$, $\gamma =0.1$, $\lambda =0.4$, $\beta =0.5$, ${\lambda }_{T}=20.0$, ${\lambda }_{c}=8.0$, Pr = 7.0, Sc = 0.2.

The current section is devoted for the detailed discussion about the physical behavior of the unknown physical quantities such as velocity profile $U$, temperature distribution $\theta$ and mass concentration $\phi$ for several values of the appeared pertinent parameters in the flow model. The physical parameters involved in the flow model are body shape parameter $n$, mixed convection parameter ${\lambda }_{T}$, modified mixed convection parameter ${\lambda }_{C}$, Prandtle number Pr, exothermic parameter $\beta$, chemical reaction parameter ${\lambda }^{2}$, temperature relative parameter $\gamma$, activation energy parameter $E$, and Schmidt number Sc for which the numerical results of considered properties are computed and demonstrated graphically in Figs. 2, 3, 4, 5, 6, 7 and 8.

Figure 2a–c highlighted the physical behavior of profiles of velocity, temperature and concentration for various values of ${\lambda }_{T}$ keeping other dimensionless parameters fixed. The increase of mixed convection parameter clearly leads to significant increase in velocity profile and decrease in temperature distribution and mass concentration observed. It is interesting to note that the maximum values of velocity distribution at ${\lambda }_{T}=50.0$, temperature distributionat ${\lambda }_{T}=1.0$ and mass concentrationat ${\lambda }_{T}=1.0$ are achieved, and the obtained graphical results are satisfying the given boundary conditions at the surface and far from the surface in asymptotic manner. The fact is that the an increase ${\lambda }_{T}$ increase the buoyancy force which effect the speed of the fluid flow due to the ratio of temperature difference and inertial force. The graphical illustration of the above said properties for several values of modified mixed convection parameter ${\lambda }_{c}$ is presented in Fig. 3a–c. It is pertinent to highlight that as ${\lambda }_{c}$ is enhanced the velocity of the fluid reduces but opposite behavior for temperature and mass concentration is observed. The noted behavior of velocity was expected because as ${\lambda }_{C}$ is increased diffusion force increases which causes friction in velocity field. At ${\lambda }_{c}=0.1,{\lambda }_{c}=0.5$ and ${\lambda }_{c}=0.5$ the highest magnitudes for velocity distribution, temperature distribution and concentration profile are noted.

In Fig. 4a–c the effects of increasing values of body shape parameter $n$ on the distribution of velocity and temperature along with mass concentration profile are highlighted. We can note from the obtained results that the decrease in velocity profile $U$ but reverse mechanisms for temperature and mass concentration are observed.

The impact of different values of Pr on velocity profile, temperature and mass concentration are shown in Fig. 5a–c. It is concluded from the geometrical interpretation that velocity and temperature of the flow domain slow down but mass concentration is improved gradually.

Figure 6a–c, we have shown the variation of the corresponding velocity field, temperature distribution and mass concentration for several values of exothermic parameter $\beta$. These functions are greatly influenced by the exothermic parameter $\beta$, as $\beta$ is increased, the velocity profile is maximum for $\beta =0.1$ and minimum for $\beta =0.3$, where the temperature and mass concentration are increased gradually.

The influence of temperature relative parameter $\gamma$ on the velocity, temperature field and mass concentration is shown in Fig. 7a–c. We see that the velocity profile is maximum for $\gamma =0.05,$ and this parameter shows its constant behavior for $\gamma$ = 0.03, 0.05 in the case of temperature field and mass concentration. Finally, the variation of the velocity, temperature field and mass concentration with activation energy parameter *E* is illustrated in Fig. 7a–c. It can be seen that the velocity exceed for *E* = 0.05, and slight changes is noted for values of *E* in the case of mass concentration. Table 2 represents the effects of different values of mixed convection parameter ${\lambda }_{T}$, it is noted that for the increasing values of this parameter the skin friction is increased and rate of heat and mass transfer are decreased. Table 3 highlights the effect of body shape parameter *n*, it is observed that skin friction is maximum for *n* = 0.3 but rate of heat and mass transfer are maximum at *n* = 02. Table 4 shows the effects of different values of activation parameter *E*, it is estimated that the skin friction, rate of heat and mass transfer are increased simultaneously.

**Table 2:** Numerical results of skin friction $\left( {\frac{{\partial {\text{U}}}}{{\partial {\text{Y}}}}} \right)_{{{\text{Y}} = 0}}$, rate of heat transfer $\left( {\frac{{\partial \uptheta }}{{\partial {\text{Y}}}}} \right)_{{{\text{Y}} = 0}}$ and rate of mass transfer $\left( {\frac{{\partial \upphi }}{{\partial {\text{Y}}}}} \right)_{{{\text{Y}} = 0}}$ for different values of $\uplambda _{{\text{T}}}$ when Pr = 7.0, n = 0.3, $\uplambda _{{\text{c}}} = 0.6$, $\upgamma = 0.1$, $\upbeta = 0.5$, $\uplambda = 0.3,{\text{E}} = 0.4$.

${\lambda }_{T}$	${\left(\frac{\partial U}{\partial Y}\right)}_{Y=0}$	$\left( {\frac{{\partial \Theta }}{{\partial Y}}} \right)_{{Y = 0}}$	${\left(\frac{\partial \phi }{\partial Y}\right)}_{Y=0}$
0.1	0.34732	0.71620	0.47028
0.5	0.43264	0.31496	0.24396
0.7	0.69661	0.27928	0.22478
1.0	0.97277	0.26765	0.22181

**Table 3:** Numerical results of skin friction $\left( {\frac{{\partial {\text{U}}}}{{\partial {\text{Y}}}}} \right)_{{{\text{y}} = 0}}$, rate of heat transfer $\left( {\frac{{\partial \uptheta }}{{\partial {\text{Y}}}}} \right)_{{{\text{Y}} = 0}}$ and rate of mass transfer $\left( {\frac{{\partial \upphi }}{{\partial {\text{Y}}}}} \right)_{{{\text{Y}} = 0}}$ for different values of n when Pr = 7.0, $\uplambda _{{\text{T}}} = 10.0$, $\uplambda _{{\text{c}}} = 0.6$, $\upgamma = 0.1$, $\upbeta = 0.5$, $\uplambda = 0.3$, E = 0.4.

$n$	${\left(\frac{\partial U}{\partial Y}\right)}_{Y=0}$	$\left( {\frac{{\partial \Theta }}{{\partial Y}}} \right)_{{Y = 0}}$	${\left(\frac{\partial \phi }{\partial Y}\right)}_{Y=0}$
0.1	2.16301	1.35363	0.78082
0.2	0.67138	5.63957	2.41498
0.3	3.98777	0.29443	0.29284
0.4	1.57465	2.04652	1.07511
0.5	3.85909	1.31551	0.65999

**Table 4:** Numerical results of skin friction $\left( {\frac{{\partial {\text{U}}}}{{\partial {\text{Y}}}}} \right)_{{{\text{y}} = 0}}$, rate of heat transfer $\left( {\frac{{\partial \uptheta }}{{\partial {\text{Y}}}}} \right)_{{{\text{Y}} = 0}}$ and rate of mass transfer $\left( {\frac{{\partial \upphi }}{{\partial {\text{Y}}}}} \right)_{{{\text{Y}} = 0}}$ for different values of E when Pr = 7.0, $\uplambda _{{\text{T}}} = 10.0$, $\uplambda _{{\text{c}}} = 0.6$, $\upgamma = 0.1$, $\upbeta = 0.5$, $\uplambda = 0.3$, n = 0.3.

E	${\left(\frac{\partial U}{\partial Y}\right)}_{Y=0}$	$\left( {\frac{{\partial \Theta }}{{\partial Y}}} \right)_{{Y = 0}}$	${\left(\frac{\partial \phi }{\partial Y}\right)}_{Y=0}$
0.02	0.20204	0.92421	0.15766
0.04	0.33598	1.95546	0.68821
0.05	0.50400	1.88940	0.71208

## Conclusions

The numerical simulations have been conducted in order to examine the effects of different parameters involved in the flow model on some pertinent quantities that is velocity profile, temperature distribution, mass concentration, skin friction, heat and mass transfer along the curved surface by using finite difference method. The main findings are summarized as follows.

- The increase in ${\lambda }_{T}$ increase the buoyancy force like pressure gradient thus the velocity profile is increased for highest value of ${\lambda }_{T}=50.0$, temperature profile, mass concentration are maximum for lower value ${\lambda }_{T}=1.0$. In addition, It is observed that skin friction increased, heat and mass transfer are decreased for increased values of ${\lambda }_{T}$.
- The increase in ${\lambda }_{c}$ enhance the ratio mass concentration difference to dynamic viscosity which reduced the velocity of the fluid but opposite trend for temperature and mass concentration has observed.
- For increasing values of body shape parameter $n$ the velocity of the fluid is decreased and reverse mechanism for temperature profile and mass concentration has noted due to tangential effects of gravity. On the other hands, very interesting behavior for skin friction, heat and mass transfer have noted; the skin friction is maximum at *n* = 0.3, the heat and mass transfer are maximum for *n* = 0.2.
- The velocity profile, temperature distribution and mass concentration are significantly influenced by the exothermic parameter $\beta$, as $\beta$ is increased, the velocity profile is maximum for $\beta =0.1$ and minimum for $\beta =0.3$, where the temperature and mass concentration are increased gradually.
- It is observed that the velocity profile is maximum for $\gamma =0.05,$ and this parameter shows its constant behavior for $\gamma$ = 0.03, 0.05 in the case of temperature field and mass concentration.
- It can be seen that the velocity exceed for energy activation parameter *E* = 0.05, and slight changes is noted for the case of temperature distribution and mass concentration. Further, it is very interesting to note that magnitude of skin friction, rate of heat and mass transfer are increased very prominently.

## References

[1] Pop I, Takhar HS (2002). Free convection from a curved surface. J. Appl. Math. Mech..

[2] Magyari E, Pop I, Keller B (2002). A note on the free convection from curved surfaces. ZAMM J. Appl. Math. Mech./Zeitschriftfür Angewandte Mathematik and Mechanik.

[3] Maleque K (2013). Effects of exothermic/endothermic chemical reactions with Arrhenius activation energy on MHD free convection and mass transfer flow in presence of thermal radiation. J. Thermodyn..

[4] Bhattacharyya K, Mukhopadhyay S, Layek GC (2013). Similarity solution of mixed convective boundary layer slip flow over a vertical plate. Ain Shams Eng. J..

[5] Bachok N, Ishak A, Pop I (2013). Mixed convection boundary layer flow over a moving vertical flat plate in an external fluid flow with viscous dissipation effect. PLoS ONE.

[6] Hayat T, Shaheen U, Shafiq A, Alsaedi A, Asghar S (2015). Marangoni mixed convection flow with Joule heating and nonlinear radiation. AIP Adv..

[7] Seshadri R, Munjam SR (2016). Mixed convection flow due to a vertical plate in the presence of heat source and chemical reaction. Ain Shams Eng. J..

[8] Ashraf M, Fatima A, Gorla RSR (2017). Periodic momentum and thermal boundary layer mixed convection flow around the surface of a sphere in the presence of viscous dissipation. Can. J. Phys..

[9] Okechi NF, Jalil M, Asghar S (2017). Flow of viscous fluid along an exponentially stretching curved surface. Results Phys..

[10] Hayat T, Saif RS, Ellahi R, Muhammad T, Ahmad B (2017). Numerical study of boundary-layer flow due to a nonlinear curved stretching sheet with convective heat and mass conditions. Results Phys..

[11] Ashraf M, Ahmad U, Chamkha AJ (2019). Computational analysis of natural convection flow driven along curved surface in the presence of exothermic catalytic chemical reaction. Comput. Therm. Sci..

[12] Dhlamini M, Kameswaran PK, Sibanda P, Motsa S, Mondal H (2019). Activation energy and binary chemical reaction effects in mixed convective nanofluid flow with convective boundary conditions. J. Comput. Design Eng..

[13] Abbas A, Ashraf M, Chu YM, Zia S, Khan I, Nisar KS (2020). Computational study of the coupled mechanism of thermophoretic transportation and mixed convection flow around the surface of a sphere. Molecules.

[14] Abbas A, Muhmmad A (2020). Combined effects of variable viscosity and thermophoretic transportation on mixed convection flow around the surface of a sphere. Therm. Sci..

[15] Ashraf M, Abbas A, Ali A, Shah Z, Alrabaiah H, Bonyah E (2020). Numerical simulation of the combined effects of thermophoretic motion and variable thermal conductivity on free convection heat transfer. AIP Adv..

[16] Ashraf M, Abbas A, Zia S, Chu YM, Khan I, Nisar KS (2020). Computational analysis of the effect of nano particle material motion on mixed convection flow in the presence of heat generation and absorption. CMcC Comput. Mater. Continua.

[17] Alghamdi M (2020). Significance of arrhenius activation energy and binary chemical reaction in mixed convection flow of nanofluid due to a rotating disk. Coatings.

[18] Ahmad U, Ashraf M, Khan I, Nisar KS (2020). Modeling and analysis of the impact of exothermic catalytic chemical reaction and viscous dissipation on natural convection flow driven along a curved surface. Therm. Sci..

[19] Ullah Z, Ashraf M, Zia S, Chu Y, Khan I, Nisar KS (2020). Computational analysis of the oscillatory mixed convection flow along a horizontal circular cylinder in thermally stratified medium. CMC Comput. Mater. Continua.

[20] Chaudhary S, Kanika KM (2020). Viscous dissipation and Joule heating in MHD Marangoni boundary layer flow and radiation heat transfer of Cu–water nanofluid along particle shapes over an exponential temperature. Int. J. Comput. Math..

[21] Krishna MV, Jyothi K, Chamkha AJ (2020). Heat and mass transfer on MHD flow of second-grade fluid through porous medium over a semi-infinite vertical stretching sheet. J. Porous Media.

[22] Islam S, Jawad M, Gokul KC, Zubair M, Alrabaiah H, Shah Z (2020). Entropy optimization in MHD nanofluid flow over a curved exponentially stretching surface with binary chemical reaction and Arrhenius activation energy. J. Phys. Commun..

[23] Muhammad R, Khan MI, Jameel M, Khan NB (2020). Fully developed Darcy–Forchheimer mixed convective flow over a curved surface with activation energy and entropy generation. Comput. Methods Programs Biomed..

[24] Khan MI, Qayyum S, Kadry S, Khan WA, Abbas SZ (2020). Theoretical investigations of entropy optimization in electro-magneto nonlinear mixed convective second order slip flow. J. Magnet..

[25] Hayat T, Khan SA, Alsaedi A, Zai QZ (2020). Computational analysis of heat transfer in mixed convective flow of CNTs with entropy optimization by a curved stretching sheet. Int. Commun. Heat Mass Transfer.

